# Etiquetado de alimentos en Ecuador: implementación, resultados y acciones pendientes

**DOI:** 10.26633/RPSP.2017.54

**Published:** 2017-05-15

**Authors:** Adrián Alberto Díaz, Paula Mariana Veliz, Gabriela Rivas-Mariño, Carina Vance Mafla, Luz María Martínez Altamirano, Cecilia Vaca Jones

**Affiliations:** 1 Organización Panamericana de la Salud Organización Panamericana de la Salud Quito Ecuador Organización Panamericana de la Salud, Quito, Ecuador.; 2 Pontificia Universidad Católica del Ecuador Pontificia Universidad Católica del Ecuador Quito Ecuador Pontificia Universidad Católica del Ecuador, Quito, Ecuador.; 3 Gestión Interna de Promoción de la Nutrición Seguridad y Soberanía Alimentaria, Ministerio de Salud Pública Quito Ecuador Gestión Interna de Promoción de la Nutrición, Seguridad y Soberanía Alimentaria, Ministerio de Salud Pública, Quito, Ecuador.; 4 Instituto Suramericano de Gobierno en Salud Río de Janeiro Brasil Instituto Suramericano de Gobierno en Salud, Río de Janeiro, Brasil.; 5 Control Sanitario Ministerio de Salud Pública Quito Ecuador Control Sanitario, Ministerio de Salud Pública, Quito, Ecuador; 6 Bernard van Leer Foundation Bernard van Leer Foundation La Haya Holanda Bernard van Leer Foundation, La Haya, Holanda.

**Keywords:** Etiquetado de alimentos, políticas de salud, Ecuador, Food labeling, public health policy, Ecuador

## Abstract

Las enfermedades no transmisibles representan la principal causa de muerte en el mundo entero, siendo responsables de 38 millones de las defunciones registradas en 2012. Esta epidemia se asocia, principalmente, al tabaquismo, al consumo excesivo de alcohol, el sedentarismo y cambios en el patrón alimentario, caracterizado por el consumo de dietas con un elevado contenido de azúcar y grasas saturadas, propio de los alimentos procesados y bebidas azucaradas, sumado a una escasa ingesta de frutas y hortalizas. El Ecuador no escapa a ese perfil epidemiológico ni a los cambios en el patrón de consumo de alimentos, por lo cual, el Estado Ecuatoriano diseñó e implementó un plan de acción orientado a modificar el entorno obesogénico, que contempla seis líneas estratégicas, una de las cuales es la implementación de un sistema de etiquetado nutricional tipo semáforo a los alimentos procesados, a finales de 2014, orientado a garantizar el derecho de las personas a la información oportuna, clara, precisa y no engañosa sobre el contenido y características de estos alimentos. El presente artículo analiza el proceso de implementación del etiquetado de alimentos procesados, los resultados alcanzados hasta la fecha y propone medidas complementarias que se requieren para el logro de la meta prevista en el Plan Nacional del Buen Vivir, a la luz de la nueva evidencia científica y los distintos acuerdos y marcos regulatorios disponibles en nuestra Región. La metodología de estudio incluyó revisión bibliográfica y de actas, entrevistas a informantes clave, y análisis y procesamiento de fuentes secundarias.

## Abstract (en)

Noncommunicable diseases are the world’s leading cause of death, responsible for 38 million deaths in 2012. This epidemic is primarily associated with smoking, excessive alcohol consumption, sedentary lifestyle, and changes in dietary patterns, characterized by diets high in sugar and saturated fat, typical of processed foods and sugar-sweetened beverages, plus low intake of fruits and vegetables. Ecuador is no exception to this epidemiological profile or to changes in eating patterns. Thus, Ecuador’s government designed and implemented an action plan aimed at changing the obesogenic environment, which includes six strategic lines. One of these is implementation of a traffic-light nutritional labeling system for processed foods, in late 2014, aimed at guaranteeing people’s right to timely, clear, accurate, and non-deceptive information on the content and characteristics of these foods. This article analyzes implementation of processed food labeling and results to date, and proposes complementary measures needed to reach the goal in the National Plan for Good Living, in light of new scientific evidence and different agreements and regulatory frameworks in our Region. Methods included a literature and documentary review, key informant interviews, and analysis and processing of secondary sources.

La epidemia de enfermedades no transmisibles (ENT) que afrontan todos los países del mundo es de tal magnitud que, en septiembre de 2011, el Secretario General de las Naciones Unidas convocó una reunión de Alto Nivel para tratar este problema de salud pública. Resultado de ello es la Declaración de Alto Nivel sobre la Prevención y el Control de las ENT (1), donde los Jefes de Estado reconocen que estas enfermedades “constituyen unos de los principales obstáculos para el desarrollo en el siglo XXI, que socavan el desarrollo social y económico en todo el mundo y ponen en peligro la consecución de los objetivos de desarrollo convenidos internacionalmente”. Asimismo, la declaración incluye un conjunto de medidas que deben ser adoptadas para luchar contra esta epidemia.

Según la Organización Mundial de la Salud (OMS), las ENT son la principal causa de muerte en el mundo y responsables de 38 millones (68%) de las defunciones registradas en 2012, de las cuales, 16 millones (40%) se producen antes de los 70 años de edad (2). Un dato alentador es que en la última década se ha registrado una disminución constante (12%) de la tasa de mortalidad por ENT estandarizada por edad (3). En el mismo período, la probabilidad de muerte prematura por ENT se ha reducido 15% en el mundo, siendo los países de altos ingresos los que exhiben los mayores progresos gracias a la reducción de la mortalidad por enfermedades cardiovasculares (ECV) asociada a la disminución de la tasa de hipertensión arterial, el menor consumo de tabaco y los avances en el tratamiento médico de los trastornos hipertensivos (2, 3).

Respecto de la prevalencia de obesidad se observa un aumento de más del doble entre 1980 y 2014, afectando y en la actualidad afecta a 11% de los hombres y 15% de las mujeres mayores de 18 años en todo el mundo, cifra que asciende a 38 y 40%, respectivamente, en el caso del sobrepeso. En la Región de las Américas las prevalencias de sobrepeso y obesidad en ambos sexos alcanzan 61 y 27%, respectivamente (2). En menores de 5 años, estas prevalencias han ascendido de manera constante en todo el mundo durante la última década y se estima que el número de niños afectados alcanzó los 42 millones en 2013 (4). En América Latina, 7% de los menores de 5 años (3,8 millones) presenta sobrepeso u obesidad, y estos valores aumentan progresivamente durante la edad escolar y la adolescencia (4). También la prevalencia de diabetes ha experimentado un crecimiento constante en los últimos años y actualmente se estima que afecta a 9% de la población mundial (2).

Ecuador se encuentra ante el desafío de la doble carga de la malnutrición (5). En los menores de 5 años, la anemia y el retraso del crecimiento constituyen los principales problemas de salud pública, cuyas prevalencias son 25,7 y 25,3%, respectivamente, mientras que el sobrepeso y obesidad alcanzaron 8,6% en 2012. En los escolares de 5 a 11 años, la prevalencia llega a 29,9% y en los en adultos de 20 a 60 años, a 62,8% (6). Además, 4 de las 5 principales causas de muerte en Ecuador están asociadas con las ENT, que representan 31,11% del total de muertes en el país (7).

Los principales factores de riesgo de las ENT son el tabaquismo, el consumo excesivo de alcohol y las dietas con un elevado contenido de azúcar y grasas saturadas, sumados a una escasa ingesta de frutas y hortalizas y al sedentarismo (8).

Las últimas décadas se han caracterizado por un cambio en los hábitos alimentarios a escala mundial, que consiste en la sustitución progresiva de los alimentos naturales o mínimamente procesados, con alto contenido de carbohidratos complejos, fibra y micronutrientes, por alimentos procesados, energéticamente densos o ricos en grasas, sal o azúcares simples (9, 10). Evidencia de ello es que las ventas de productos procesados en el mundo han aumentado 43,7% entre el 2000 y el 2013, y en América Latina la venta de alimentos procesados y bebidas azucaradas, 48%. Asimismo, en Ecuador, el consumo per capita de dichos productos lo hizo 19,8% entre el 2000 y 2013, pasando de 73,4 kg per cápita a 87,9 kg en 2013 (10).

Uno de los determinantes que explica esta tendencia es la agresiva estrategia de mercadeo que utiliza la industria de alimentos procesados y bebidas azucaradas cuyos principales destinatarios son los niños y los adolescentes (11). En este contexto, el Plan Nacional del Buen Vivir (PNBV) de Ecuador propone “revertir la tendencia de la incidencia de obesidad y sobrepeso en niños/as de 5 a 11 años” (12), para lo cual se implementó un plan de acción orientado a modificar el entorno obesogénico a partir de un conjunto de líneas estratégicas, como la regulación del etiquetado de alimentos procesados, la promoción intensiva de la lactancia materna, la promoción de la alimentación saludable en escuelas y colegios, medidas fiscales y la regulación de publicidad dirigida a niños y adolescentes (13).

El objetivo del presente artículo es analizar el proceso de implementación del etiquetado de alimentos procesados en Ecuador, los resultados alcanzados y las medidas complementarias requeridas para alcanzar la meta prevista en el PNBV.

## MATERIALES Y MÉTODOS

Para realizar este estudio se realizó una revisión bibliográfica narrativa no sistemática que incluyó artículos publicados en revistas médicas, actas, informes y otros documentos oficiales, además de información obtenida a través de consultas a funcionarios y exfuncionarios que participaron en el proceso de implementación de la medida regulatoria, así como fuentes secundarias procedentes de estudios de mercado.

## RESULTADOS

### Implementación y situación actual

**Implementación del sistema gráfico de etiquetado de alimentos procesados**. En 2012, el Ministerio de Salud Pública (MSP), conjuntamente con el Ministerio Coordinador de Desarrollo Social (MCDS) y la Agencia Nacional de Regulación, Control y Vigilancia Sanitaria (ARCSA), convocaron a distintos actores sociales para debatir la propuesta de reglamento para el etiquetado gráfico de alimentos procesados y bebidas azucaradas (en adelante “el etiquetado”) (14). Paralelamente, se impulsó un proceso de validación (15), que evaluó la comprensión, aceptación y funcionalidad de diferentes propuestas, lo que orientó la decisión de utilizar una etiqueta semaforizada con barras horizontales, letras grandes y sin mensajes extras que pudieran confundir al consumidor. Posteriormente, en noviembre de 2013, se publicó la primera versión del Reglamento Sanitario de Etiquetado de Alimentos Procesados. Finalmente, en agosto de 2014, se aprobó el denominado “Reglamento Sanitario Sustitutivo de Alimentos Procesados para el Consumo Humano 5103” (16) —actualmente en vigencia—, que amplió los plazos iniciales establecidos para su implementación.

**FIGURA 1:**
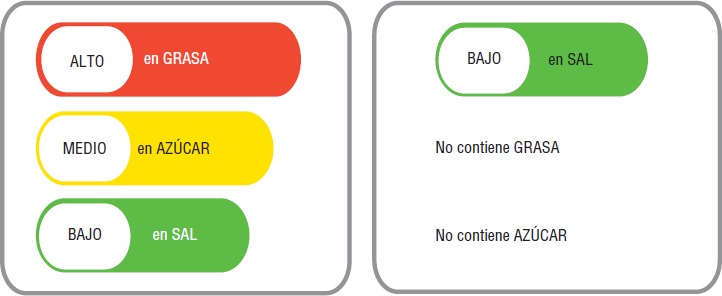
Sistema gráfico del etiquetado de alimentos procesados de Ecuador

El sistema gráfico previsto por el reglamento establece una barra roja para los productos con contenido “ALTO” en grasa, azúcar o sal, la barra de color amarillo, para el contenido “MEDIO”, y la barra de color verde, para el contenido “BAJO” en estos componentes (16) (figura 1). Los puntos de corte (cuadro 1) se fijaron mediante el cálculo de la cantidad en gramos que el producto contiene (azúcar, grasa o sal) conforme a la recomendación de la Organización Panamericana de la Salud (OPS) (17), a pesar de que la industria proponía realizar un cálculo basado en porcentajes.

Para informar sobre el correcto uso y los beneficios del etiquetado, el MSP desarrolló una campaña de comunicación en radio, televisión y otros medios de comunicación. Por su parte, la ARCSA desplegó una campaña nacional dirigida a la industria con el fin de explicar la aplicación del reglamento y creó una página web para responder dudas o inquietudes, que incluye un simulador para visualizar gráficamente eletiquetado cuando se introduce la composición de un producto.

Sin duda, todo este proceso no fue simple ni estuvo exento de fuertes negociaciones con la industria, que implicaron concesiones que modificaron la propuesta original (18), como, por ejemplo, la eliminación de la prohibición de usar imágenes o animales, la inclusión de definiciones de azucares (propias o añadidas), edulcorantes (no calóricos, naturales o artificiales) y sal, el reemplazo de valores de sal en gramos por sodio en miligramos, o la opción de ubicar el etiquetado en el panel principal o secundario, a pesar de que las pruebas científicas disponibles muestras que el etiquetado frontal contribuye a identificar y seleccionar los alimentos saludables (19).

Uno de los elementos de debate y análisis en este proceso fue el tipo de instrumento legal que se utilizaría para la implementación de esta medida. A pesar de que existía la posibilidad de hacerlo mediante una ley, lo cual daría mayor robustez a la disposición regulatoria, se consideró también que este proceso sería más lento y complejo, por lo cual se optó por implementar el etiquetado mediante un acuerdo ministerial,que se incluiría posteriormente en el nuevo Código de Salud pendiente de aprobación por la Asamblea Nacional. Hasta que ello ocurra, el etiquetado está en permanente riesgo de eliminación debido a constantes presiones de distintos actores, principalmente el sector empresarial local y transnacional.

Como ejemplo de estas presiones cabe citar que —durante el Comité de Obstáculos Técnicos al Comercio de la Organización Mundial de Comercio (OMC)— el representante de la Unión Europea recordó a Ecuador que el Codex sobre Etiquetado Nutricional no ha establecido niveles mínimos para los nutrientes abarcados por el reglamento. Además, sugirió que “*se suspenda la aplicación de la medida para que las empresas dispongan de un plazo de, al menos, seis meses para adaptarse a los nuevos requisitos*” (20). En ese mismo Comité, el representante de los Estados Unidos de América señaló que Ecuador “*no llevó a cabo ninguna consulta pública con las partes nacionales interesadas y los interlocutores comerciales de la OMC*”, una afirmación imprecisa si se tienen en cuenta los informes previamente citados. Asimismo, México emitió una declaración ante la OMC en la cual plantea dudas y solicita un conjunto de modificaciones al reglamento RTE INEN 022, que coincidían con la posición expresada por la industria de alimentos (21). Estas observaciones fueron oportuna y adecuadamente respondidas por el Gobierno Ecuatoriano, de manera que no afectaron la continuidad de la medida.

**Fuente:** referencia 16.

**A un año de la implementación del etiquetado**. A fines de 2015, el MSP impulsó una evaluación del etiquetado con el objetivo de conocer la percepción, el uso y la comprensión por parte de los consumidores (18). El método del estudio incluyó grupos focales de consumidores, entrevistas en profundidad a informantes clave del sector productivo y comercial y observación estructurada para analizar los reglamentos vigentes y los empaques de los alimentos procesados. El principal hallazgo de la investigación fue que el sistema gráfico “semáforo” es ampliamente reconocido y comprendido por parte de los consumidores, quienes consideran que brinda información útil e importante.

Al comparar el sistema gráfico con una etiqueta alternativa de valor diario recomendado (VDR) o GDA (por sus siglas en inglés), los entrevistados consideraron a esta última menos comprensible que el sistema gráfico. También se observó que los consumidores utilizaron diferentes estrategias de adaptación o compensación, entre las cuales destacan: dejar de consumir productos con etiquetado que indica contenido “alto”; aumentar el consumo de productos con edulcorantes artificiales; optar por productos con etiquetado que indica contenido “medio” y “bajo”, y consumir en menor cantidad o frecuencia los productos con contenido “alto”. Además, el análisis de los empaques puso en evidencia el incumplimiento o libre interpretación del etiquetado por parte de algunas empresas.

**CUADRO 1:** Contenido de componentes y concentraciones permitidas por la Organización Panamericana de la Salud para los alimentos procesados que contienen grasas, azúcares y sal

Nivel/Componentes	Concentración “Baja”	Concentración “Media”	Concentración “Alta”
Grasas totales	≤ 3 g en 100 g	> 3 g a < 20 g en 100 g	≥ 20 g en 100 g
	≤ 1,5 g en 100 mL	> 1,5g a < 10 en 100 mL	≥ 10 g en 100 mL
Azucares	≤ 5 g en 100 g	> 5 g a < 15 g en 100 g	≥ 15 g en 100 g
	≤ 2,5 g en 100 mL	> 2,5 g a < 7,5 g en 100 mL	≥ 7,5 g en 100 mL
Sal (sodio)	≤ 120 mg de sodio en 100 g	> 120 g a < 600 mg de sodio en 100 g	≥ 600 mg de sodio en 100 g
(sustituido por el Art. 3 del Acdo. 00004832, R.O. 237-S, 2-V-2014)	≤ 120 mg de sodio en 100 mL	> 120 g a < 600 mg de sodio en 100 mL	≥ 600 mg de sodio en 100 mL

***Fuente:*** referencia 3.

Contrariamente a la opinión de los consumidores, los representantes de la industria alimentaria consideraron que la información no es útil ni de interés para la población y que la alternativa GDA es mejor que el sistema gráfico. También manifestaron su disconformidad con los plazos establecidos para la implementación, destacaron el impacto sobre las ventas de algunos productos y desestimaron el efecto de esta medida para reducir el sobrepeso y la obesidad por considerar que los productos procesados representan una mínima proporción de la dieta de los ecuatorianos.

**FIGURA 2:**
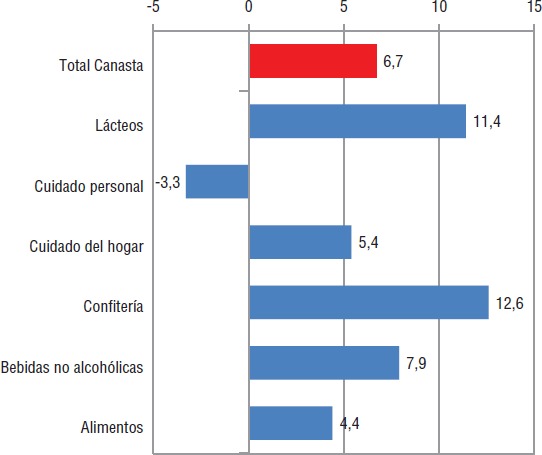
Comportamiento de la «Canasta Nielsen» en Ecuador, total y por componente,entre enero y octubre de 2014 y enero y octubre de 2015

Simultáneamente, se realizó un análisis de mercado para la categoría jugos y gaseosas a fin de conocer el comportamiento de estos productos antes y después de la implementación del etiquetado (22). El estudio se basó en una muestra representativa de 2 600 establecimientos comerciales distribuidos en 49 ciudades de la sierra y la costa, que representan 56% de la población ecuatoriana y 87% de la población urbana de todo el país. Entre enero y octubre de 2014 y enero-octubre de 2015, se observó que la canasta total productos estudiados (integrada por bebidas no alcohólicas, confitería, cuidado personal, alimentos, cuidado del hogar y lácteos) creció 6,7%. Las categorías que más crecieron fueron: confitería 12,6%, lácteos 11,4% y bebidas no alcohólicas 7,9%, esta última impulsada por el crecimiento de aguas, isotónicas y té helado-polvo (RTD) (figura 2). Asimismo, las gaseosas crecieron 5,9% en cantidad de litros y 3,7% en volumen de ventas, mientras que el precio promedio por litro se redujo 1,9%, lo cual se explicaría por la opción de los consumidores por segundas marcas más económicas que las que lideran el mercado. Además, hubo un crecimiento de 47,6%, en la cantidad de litros de las gaseosas “light”, las cuales, si bien aún representan un proporción muy pequeña dentro la categoría gaseosas, marcarían una tendencia en las preferencias de los consumidores. El crecimiento de los jugos procesados fue mayor que el de las gaseosas (9,9% en cantidad de litros y 8,6% en volumen de negocios), mientras que el precio medio por litro se redujo 1,3%. Un dato destacable es que los néctares (40% de fruta en su composición), pierden entre 1,5 y 1,8% en la participación del mercado frente al grupo de “bebidas + refrescos” (10 a 20% de frutas).

Por otro lado, las encuestas de Euromonitor para Ecuador muestran un crecimiento positivo aunque decreciente de las bebidas no alcohólicas entre 2010 y 2015, tanto en volumen como en valor de ventas, con un estancamiento entre 2014 y 2015 (cuadro 2) (23–25).

**FIGURA 3:**
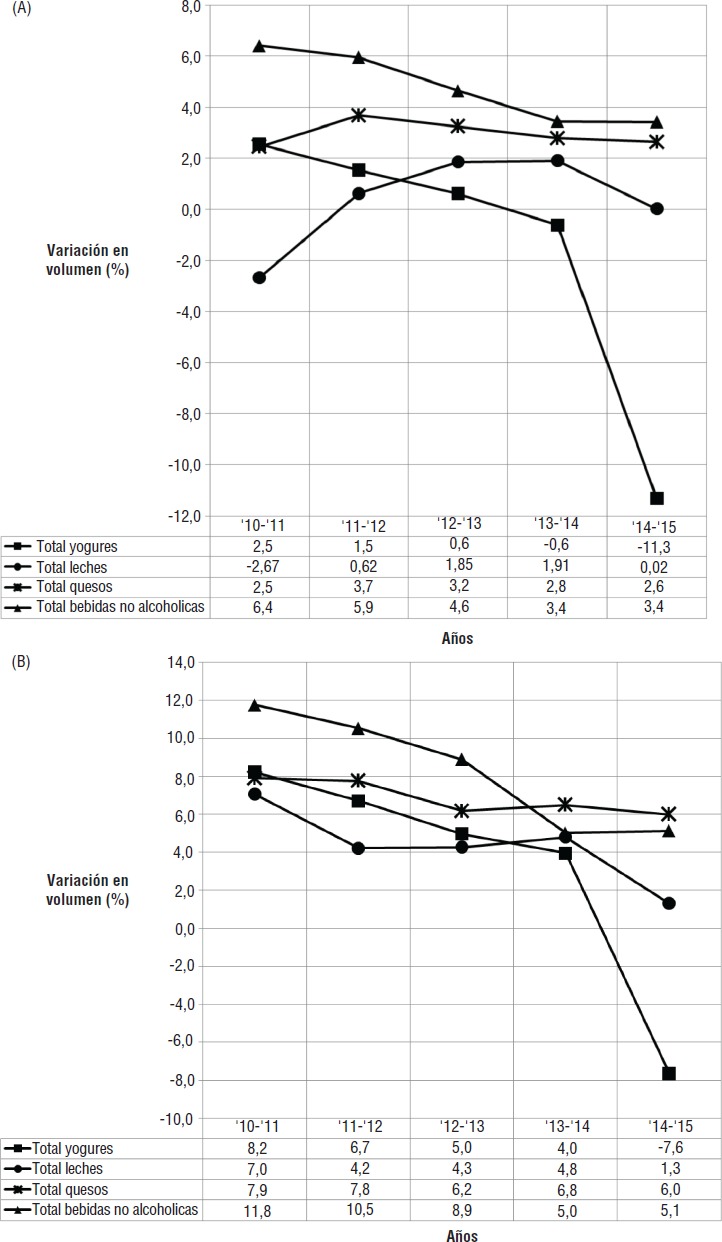
Porcentaje de variación en volumen y valor de los diferentes grupos de productos entre dos años sucesivos durante el período 2010-2015

Un análisis particular merece el mercado de los lácteos, pues representantes del sector ganadero–lácteo expresaron reiteradamente su preocupación por la caída de ventas, que atribuían a la implementación del etiquetado. Esta afirmación, sin embargo, concuerda parcialmente con la información provista por Euromonitor, que muestra que el grupo “lácteos” ha tenido un crecimiento continuo entre 2010 y 2015 en el valor de ventas, mientras que en volumen la tendencia fue positiva hasta el 2014, con un decrecimiento entre 2014 y 2015 (figura 3). Este comportamiento sería atribuible a la caída de las leches saborizadas y de algunos tipos de yogures cuyo etiquetado en rojo (por su alto contenido en azúcar) podría haber desalentado su consumo (figura 3). Se observa, también, que la medida regulatoria habría acentuado una tendencia que se venía insinuando antes de la implementación del etiquetado.

## DISCUSIÓN

La evidencia científica es cada vez más firme respecto a la asociación entre la epidemia de ENT y el cambio del patrón alimentario. No obstante, la industria sigue negando tal asociación, se resiste a adoptar las medidas regulatorias de los estados e instan a las asociaciones nacionales a organizarse en contra de cualquier iniciativa en este ámbito (26), lo cual representa un gran reto para la salud pública (27).

Frente a ello, los países de la Región cuentan con nuevos instrumentos que respaldan y alientan las medidas regulatorias por parte de los estados, como el Plan de Acción para la prevención de la obesidad en la niñez y adolescencia 2014-2019 y el Modelo de Perfil de Nutrientes de la OPS (28). Además, las experiencias de Bolivia, Chile y México, que han aprobado leyes para regular el etiquetado de alimentos procesados (29, 30) o aplican impuestos a las bebidas azucaradas (31), constituyen importantes antecedentes para otros países de la Región.

***Fuente:*** Informe Nielsen, 2015. Retail Index.

La experiencia de Ecuador ha sido ampliamente reconocida (32), ya que brinda información clara a los consumidores y ha promovido que la industria alimentaria modifique la composición de algunos de sus productos o los retire del mercado (18). No obstante, el estudio cualitativo realizado en 2015, el nuevo Perfil de Nutrientes de la OPS y los análisis de consumo de alimentos procesados y bebidas azucaradas en Ecuador señalan la necesidad de revisar el etiquetado y acompañarlo de medidas complementarias con el fin de revertir la tendencia en el patrón alimentario de la población.

**CUADRO 2:** Comportamiento del mercado de lácteos en Ecuador según el tipo de producto y de bebidas azucaradas, 2010-2015

Producto	Variables	Periodo					
		2010	2011	2012	2013	2014	2015
Quesos procesados	Volumen (toneladas)	2 740,00	2 830,00	2 893,57	2 965,30	3 009,47	3 042,70
	Ventas (millones de $US)	22,39	24,08	25,56	26,92	28,48	29,83
Quesos no	Volumen (toneladas)	11 120,00	11 370,00	11 828,79	12 235,30	12 614,28	12 993,06
procesados	Ventas (millones de $US)	92,34	99,71	107,83	114,70	122,35	130,04
Total quesos	Volumen (toneladas)	13 860,00	14 200,00	14 722,35	15 200,60	15 623,74	16 035,77
	Ventas (millones de $US)	114,73	123,79	133,39	141,63	150,83	159,87
Leches saborizadas	Volumen (toneladas)	24 175,68	24 853,69	25 117,08	26 509,44	26 744,14	23 715,17
	Ventas (millones de $US)	63 891,49	72 324,23	72 914,9	77 277,67	81 484,05	75 332
Leches naturales (*)	Volumen (toneladas)	391 496,63	379 724,21	381 988,4	388 131,6	395 816,2	398 912,54
	Ventas (millones de $US)	369 923.8	392 067,71	411 037,11	427 350,52	447 326,87	460 396,98
Total leches	Volumen (toneladas)	415,672.31	404,577.9	407,105.48	414,641.04	422,560.34	422,627.71
	Ventas (millones de $US)	433 815,29	464 391,94	483 952,01	504 628,19	528 810,92	535 728,98
Yogurt bebible	Volumen (toneladas)	44 150,99	45 277,56	45 165,14	45 118,66	44 463,97	38 169,87
	Ventas (millones de $US)	100,06	108,87	114,42	119,23	122,93	109,12
Yogurt con frutas	Volumen (toneladas)	5 390,04	5 537,50	6 106,32	6 350,80	6 549,50	6 455,33
	Ventas (millones de $US)	20,28	21,60	24,07	25,80	27,48	27,82
Yogurt natural	Volumen (toneladas)	3 797,93	3 882,26	4 266,00	4 413,30	4 526,50	4 630,74
	Ventas (millones de $US)	10,74	11,40	12,91	13,88	14,77	15,61
Total yogures	Volumen (toneladas)	53 338,97	54 697,32	55 537,46	55 882,76	55 539,97	49 255,94
	Ventas (millones de $US)	131,09	141,87	151,39	158,91	165,19	152,56
Total lácteos	Volumen (toneladas)	482 871,28	473 475,22	477 365,29	485 724,4	493 724,05	487 919,42
	Ventas (millones de $US)	434 061,11	464 657,6	484 236,79	504 928,73	529 126,94	536 041,41
Total bebidas	Volumen (Millones litros)	1 632,0	1 736,6	1 839,9	1 925,3	1 991,7	2 059,9
azucaradas	Ventas (millones de $US)	1 493,8	1 669,5	1 845,2	2 009,0	2 109,7	2 218,0

***Fuente:*** elaboración propia a partir de las referencias 23-25.

En febrero de 2016, el MSP convocó una reunión de expertos para revisar y fortalecer el quienes elaboraron un conjunto de recomendaciones entre las cuales se destacan las siguientes: 1) ajustar el etiquetado al Modelo de Perfil de Nutrientes de la OPS; 2) fortalecer la vigilancia y sancionar a las empresas que incumplan el reglamento, así como el Código Internacional de Comercialización de Sucedáneos de la Lecha Materna (33, 34); 3) regular la publicidad dirigida a niños y adolescentes; 4) implantar medidas fiscales a los alimentos y bebidas procesadas, y 5) incluir el etiquetado en alimentos que contengan grasa, sal y azúcar añadidas para lactantes.

En relación con este último punto vale la pena destacar las consecuencias negativas y duraderas que tiene exponer a los lactantes a sabores dulces, pues ello condiciona esta predilección de por vida, lo cual, a su vez, se asocia con mayor prevalencia de diabetes, sobrepeso y obesidad (35).

Respecto de medidas impositivas, es importante tener en cuenta los resultados de un reciente estudio realizado en Ecuador sobre la elasticidad-precio de la participación, la elasticidad-precio de la intensidad y la elasticidad cruzada entre bebidas azucaradas (BA) y no azucaradas (BNA). En él se demuestra que la elasticidad-precio varía entre-1,17 y -1,33 para las BA y entre -1,0 y-1,24 para las BNA; es decir, existe sensibilidad de la demanda de las BA y BNA a las variaciones de precio y, por tanto, las medidas fiscales sobre las BA o el abaratamiento de los precios de las BNA vía subsidios tendrían impacto sobre su consumo (36).

Por otro lado, en mayo de 2016, la Asamblea Nacional debatió y aprobó la Ley Orgánica para el Equilibrio de la Finanzas Pública la cual establece que “Las gaseosas con menos de 25 gramos de azúcar, y las bebidas energizantes tendrán un ICE (Impuesto a los Consumo Especiales) del 10% ad valorem, mientras que las bebidas con más de 25 gramos de azúcar por litro de bebida pagarán el ICE en función de los gramos de azúcar que contengan (impuesto específico de 0,0018 centavos de dólar por cada gramo de azúcar añadida). Se encuentran exentos los productos lácteos y sus derivados, así como el agua mineral y los jugos que tengan más de 50% de contenido natural” (37). Esta medida, si bien es positiva, ha tenido una finalidad primordialmente recaudatoria y difiere de la experiencia mexicana (31), la cual ya ha mostrado impacto en la reducción del consumo de los alimentos malsanos (38).

A pesar de la necesidad de ajustar y fortalecer el etiquetado a la luz de nuevas evidencias científicas y de los resultados de las evaluaciones realizadas, se teme que ese proceso ponga en riesgo lo alcanzado hasta la fecha, pues implicaría reabrir el debate sobre el papel regulador del Estado para garantizar la salud y la nutrición de la población, en medio de una coyuntura económica y política nacional e internacional en la que afloran con renovados ímpetus los discursos que defienden los principios del libre mercado como valor superior.Este riesgo estaría minimizado si hubiera más presencia de organizaciones de la sociedad civil comprometidas con la defensa de los consumidores, como ocurre en Chile o en México, por mencionar solo algunos ejemplos.

***Fuente:*** elaboración propia a partir de las referencias (23-25).

El análisis del proceso del Ecuador permite extraer algunas conclusiones, recomendaciones y enseñanzas que pueden ser útiles para otros países dispuestos a aplicar políticas públicas para el control de las ENT y la epidemia de sobrepeso y obesidad en la población infantojuvenil. Entre ellas destacan las siguientes: a) toda medida regulatoria afrontará una férrea oposición y presiones a todo nivel por parte de la industria de los alimentos procesados; b) una sola medida no es suficiente para desalentar el consumo de alimentos malsanos; c) el abordaje debe ser intersectorial, con la participación, no solo de los ministerios del área social, sino también de los sectores de la producción, la economía y las finanzas; d) toda medida regulatoria debe estaracompañada de una adecuada estrategia de información y comunicación, así como de mecanismos de vigilancia y sanción de posibles violaciones; e) se debe estimular activamente la participación de organizaciones de la sociedad civil como aliado clave para la implementación y vigilancia ciudadana de medidas regulatorias, y d) en Ecuador, el etiquetado debe complementarse con medidas fiscales sobre los alimentos procesados y bebidas azucaradas adoptando un enfoque de salud pública más claro, así como con la regulación de la publicidad de estos productos dirigida a niños y adolescentes de estos productos.

### Agradecimientos

Los autores agradecen a Diana Rodriguez, Mónica Quinatoa, Estefany Jarrin y Javier Muñoz sus aportaciones y comentarios a este trabajo.

### Declaración

Las opiniones expresadas por los autores son de su exclusiva responsabilidad y no reflejan necesariamente los criterios ni la política de la RPSP/PAJPH o de la OPS.
